# Effect of fasudil on experimental autoimmune neuritis and its mechanisms of action

**DOI:** 10.1590/1414-431X20198669

**Published:** 2019-12-20

**Authors:** Yanyin Zhao, Bingyou Liu, Yi Wang, Baoguo Xiao

**Affiliations:** Department of Neurology, Huashan Hospital, Fudan University, Shanghai, China

**Keywords:** Fasudil, Experimental autoimmune neuritis, Th1, Th17, Tregs, M2 macrophages polarization

## Abstract

This study aimed to investigate the therapeutic effect of fasudil on treating experimental autoimmune neuritis (EAN). Twenty-four EAN mice were randomly assigned to fasudil treatment (Fasudil group) or saline treatment (EAN model group) for 28 days. Clinical symptom score was evaluated every other day; inflammatory cell infiltration, demyelination, anti-myelin basic protein (MBP), inflammatory cytokines, inducible nitric oxide synthase (iNOS), and arginase-1 were detected in sciatic nerves at day 28. Th1, Th2, Th17, and Tregs proportions in splenocytes were detected at day 28. Clinical symptom score was found to be attenuated in the Fasudil group compared to the EAN model group from day 12 to day 28. Sciatic nerve inflammatory cell counts by HE staining and demyelination by luxol fast blue staining were both reduced, while MBP was increased in the Fasudil group compared to the EAN model group at day 28. Interferon γ (IFN-γ) and interleukin (IL)-17 were reduced, while IL-4 and IL-10 were elevated in the Fasudil group at day 28. Sciatic nerve M1 macrophages marker iNOS was decreased while M2 macrophages marker arginase-1 was increased in the Fasudil group at day 28. CD4^+^IFN-γ^+^ (Th1) and CD4^+^IL-17^+^ (Th17) cell proportions were both decreased, CD4^+^IL-4^+^ (Th2) cell proportion was similar, while CD25+FOXP3+ (Treg) cell proportion in splenocytes was increased in the Fasudil group. In summary, fasudil presented a good therapeutic effect for treating EAN by attenuating Th1/Th17 cells and promoting Tregs activation as well as M2 macrophages polarization.

## Introduction

Guillain-Barré syndrome (GBS), which is an autoimmune disease and an acute inflammatory disorder that afflicts the peripheral nervous system, is the most common and severe acute paralytic neuropathy, with approximately 100,000 persons developing this disease every year worldwide (1). GBS pathological changes are commonly characterized by the loss of peripheral nerve myelin sheath and inflammatory cell infiltration, and it often presents with symmetrical weakness of limbs, numbness, and hyporeflexia (2,3). Although great efforts have been made in early diagnosis, standardized treatment (mainly including intravenous immunoglobulin and plasmapheresis), and individualized care, there is still a proportion of GBS patients that fail to respond to treatments, resulting in poor prognosis such as long-term weakness, chronic pain, and death (4,5). Thus, it is of critical importance to explore novel treatment options for GBS.

RhoA/Rho-kinase (ROCK) is emerging as a potential therapeutic target relevant to several inflammatory neurodegenerative diseases such as multiple sclerosis, Parkinson’s disease, and Alzheimer’s disease through regulating actin organization, myosin contractility, cell cycle maintenance, cellular morphological polarization, cellular development, and transcriptional control (6–9). As a selective RhoA/ROCK inhibitor, fasudil has been clinically applied for the treatment of subarachnoid hemorrhage since 1995, and has been administered to improve the cognitive decline in stroke patients (10,11). In addition, a recent meta-analysis review observed that fasudil combined with methylcobalamin or lipoic acid promotes nerve conduction velocity in diabetic peripheral neuropathy patients (12). However, the application of fasudil in treating GBS has not yet been explored.

Our previous study found that fasudil decreased antigen-specific lymphocyte proliferation, interleukin (IL)-17 expression, interferon (IFN)-γ/IL-4 ratio, and inflammatory cell infiltration, as well as reduced damage of demyelination and axon degeneration, and finally attenuated disease severity in experimental autoimmune encephalomyelitis (EAE) mice (13). Because of the similar pathogenesis between EAE and experimental autoimmune neuritis (EAN) (a well-accepted animal model of GBS), we hypothesized that fasudil would present with good efficacy in treating EAN as well. Thus, this study aimed to investigate the therapeutic effect of fasudil on treating EAN and further explore its effect on regulating immune cells in EAN.

## Material and Methods

### EAN model construction

C57BL/6 female mice (weight: 20–22 g, age: 6–8 weeks) were purchased from Beijing Vital River Laboratory Animal Technology Co., Ltd (China). The neurogenic P0 protein peptide corresponding to amino acids 180–199 of the mouse peripheral nervous system myelin P0 protein (sequence: SSKRGRQTPVLYAMLDHSRS) was synthesized by solid-phase stepwise elongation using a Tecan/Syro peptide synthesizer (Sangon Biotech, China). EAN mouse model was constructed by immunization with 120 μg of P0 peptide 180-199 emulsified in an equal volume of Freund’s complete adjuvant (Chemicon Inc., USA) containing 0.5 mg mycobacterium tuberculosis H37Ra (Difco, USA), which was injected subcutaneously into the back of the mouse at day 0 and day 7. Furthermore, 400, 200, and 200 ng pertussis toxin (Sigma, USA) was injected subcutaneously into the tail on days –1, 0, and 3, respectively.

### Treatment

EAN mice were randomly assigned into the Fasudil group and the EAN model group (n=12 mice/group). In the Fasudil group, fasudil (Asahi Chemical Industries, Japan) was dissolved in saline to a final concentration of 4 mg/mL, and then 200 μL solution was administered per day by intraperitoneal injection from days 3 to 28 after immunization. In the EAN model group, 200 μL saline was administered per day by intraperitoneal injection from day 3 to 28 after immunization.

### Clinical symptoms assessment

Clinical symptoms score of EAN in mice was assessed at day 0, and then every other day until day 28 as follows: 0) normal; 1) reduced tonus of the tail; 2) limp tail; 3) absent of righting reflex; 4) gait ataxia; 5) mild paresis of the hind limbs; 6) moderate paraparesis; 7) severe paraparesis or paraplegia of the hind limbs; 8) tetraparesis; 9) moribund; and 10) death.

### Histopathology assessment of sciatic nerves

Mice were anesthetized and perfused with PBS and 4% buffered paraformaldehyde (Sinopharm, China) at day 28, then the sciatic nerves were obtained, fixed in 4% paraformaldehyde, and embedded in paraffin (Aladdin, China). Subsequently, 8-μm sections were cut and prepared for staining. Then, hematoxylin-eosin (HE) (Aladdin) staining was performed and inflammatory cell infiltration was assessed under a digital microscope (Olympus, Japan) and calculated as follows: 5 fields of each slide (40×) were observed, and the average number of inflammatory cells was calculated.

### Demyelination assessment of sciatic nerves

Sciatic nerve sections of mice were stained by luxol fast blue (Aladdin) to assess demyelination. Fresh sciatic nerves of mice were also obtained at day 28 and stored in liquid nitrogen, and anti-myelin basic protein (MBP) expression in sciatic nerves was determined by western blot (explained in “Western blot” subsection).

### IFN-γ, IL-4, IL-17, and IL-10 mRNA expressions in sciatic nerves

IFN-γ, IL-4, IL-17, and IL-10 mRNA expressions in sciatic nerves were determined by real-time quantitative polymerase chain reaction (RT-qPCR) (explained in “RT-qPCR” subsection).

### Inducible nitric oxide synthase (iNOS) and arginase-1 expressions in sciatic nerves

In order to assess the effect of fasudil on regulating M1 and M2 macrophages, iNOS and arginase-1 protein expressions (14) in sciatic nerves of mice were determined by western blot (see below in Western blot subsection).

### Anti-P0 peptide 180-199 immunoglobulin G (IgG) in serum

Blood samples were obtained from eye vein of mice at day 28 and serum was isolated, then serum anti-P0 peptide 180–199 IgG was determined by enzyme-linked immunosorbent assay (ELISA). In brief, purified mouse P0 180–199 was coated onto ELISA plates at 1 μg/mL in a volume of 100 μL/well. The plates were incubated overnight at room temperature and washed three times with PBS plus 0.05% Tween-20, then non-specific binding was blocked with 1* ELISA/ELISPOT Diluent (eBioscience, USA) for 1 h at room temperature. After three washings, serum samples diluted to 1:50 with 1* ELISA/ELISPOT Diluent, were applied to wells and incubated for 2 h at room temperature. Subsequently, after another five washings, biotinylated mouse anti-mouse IgG (AMS, Germany) with 1:10000 dilution was added and incubated for 2 h at room temperature. Finally, after the last five washings, the reaction was visualized with p-nitrophenyl phosphate substrate (eBioscience, USA) and read at 450 nm using an ELISA reader (BioTek, USA).

### Th1, Th2, Th17, and Tregs proportions in splenocytes

The spleen was removed under aseptic condition and splenocytes were harvested after lysing red blood cells. The effect of fasudil on regulating Th1, Th2, Th17, and Tregs in splenocytes was assessed by flow cytometry. In brief, splenocytes were double-stained with cell surface FITC-labeled CD4 (2:100 dilution) (eBioscience, USA) plus intranuclear PE-CY7-labeled IFN-γ (1:100 dilution) (eBioscience), or plus intranuclear PE-labeled IL-4 (1:100 dilution) (Biolegend, USA), or plus intranuclear APC-labeled IL-17A (1:100 dilution) (Biolegend). Splenocytes were also double-stained with cell surface APC-labeled CD25 (2:100 dilution) (eBioscience) and intercellular PE-labeled FOXP3 (5:100 dilution) (eBioscience). Then, flow cytometry was performed using Attune Nxt Flow cytometer (Thermo Fisher, USA) and analyzed using FlowJo Software Version 7.6.1 (FlowJo, USA).

### IFN-γ, IL-4, IL-17, FOXP3, and IL-10 mRNA expressions in splenocytes

IFN-γ, IL-4, IL-17, FOXP3, and IL10 mRNA expressions in splenocytes were further determined by RT-qPCR (see below).

### RT-qPCR

Total RNA was isolated from samples using Trizol reagent (Invitrogen, USA). Then, cDNA was reversely transcribed using PrimeScript TM RT Master Mix (Perfect Real Time) (TaKaRa, Japan). PCR was performed using TaKaRa TB Green TM Premix Ex Taq T on the Roche Cobas Z480 Real Time PCR system (Roche, Switzerland). GAPDH was used as internal reference and mRNA expression was calculated using the 2^-ΔΔCT^ method. The primers used in RT-qPCR are listed in Table 1.

**Table 1:** Primers used in RT-qPCR.

Gene	Forward Primer (5′-3′)	Reverse Primer (5′-3′)
IFN-γ	ATGGCTGTTTCTGGCTGTTACT	ACGCTTATGTTGTTGCTGATGG
IL-4	GCTAGTTGTCATCCTGCTCTTC	GGTGTTCTTCGTTGCTGTGAG
IL-17	TGCTGTTGCTGCTGCTGAG	TGGAACGGTTGAGGTAGTCTGA
FOXP3	CTCGCATGTTCGCCTACTTCA	TCGCTCTCCACTCGCACAA
IL-10	CTGCTAACCGACTCCTTAATGC	GCTCCACTGCCTTGCTCTTAT
GAPDH	AGGTCGGTGTGAACGGATTTG	TGTAGACCATGTAGTTGAGGTCA

### Western blot

Total protein was extracted from samples using RIPA buffer (Thermo Fisher). The protein concentration in each sample was then measured using the bicinchoninic acid (BCA) kit (Pierce Biotechnology, USA) and compared with the standard curve; the mean of two measurements was calculated for each sample. Twenty-microgram protein samples were then subjected to sodium dodecyl sulfate-polyacrylamide gel electrophoresis (SDS-PAGE) and transferred onto nitrocelulose membranes (GE, USA). After blocking with 5% skim milk for 2 h, membranes were incubated with the rabbit MBP antibody (eBioscience) with 1:1000 dilution, rabbit iNOS antibody (Becton, Dickinson and Co., USA) with 1:1000 dilution, rabbit arginase-1 antibody (Becton Dickinson and Co.) with 1:1000 dilution, and rabbit GAPDH antibody (Abcam, USA) with 1:10000 dilution overnight at 4°C. Then, membranes were incubated with goat anti-rabbit IgG conjugated to horseradish peroxidase (Transgen Biotech, China) with 1:10000 dilution for 1 h at room temperature. The bands were visualized using Novex™ ECL Chemiluminescent Substrate Reagent Kit (Invitrogen) followed by exposure to X-ray film (Kodak, USA). Then, Image J Software (Java, USA) was used to determine the density of immunoblotting results, and relative density of target protein was normalized by GAPDH density as a ratio. Then, the fold change of protein relative expressions in the Fasudil group compared to that in the EAN model group was analyzed.

### Animal ethics approval

Animal experiments in this study were approved by the Animal Ethics Committee of our Hospital, and were conducted according to the National Institutes of Health Guide for the Care and Use of Laboratory Animals and under the principles of 3R (replacing, refining, and reducing).

### Statistical analysis

Statistics was performed using SPSS 21.0 software (IBM, USA) and statistical graphs were drawn using GraphPad 6.01 software (USA). Data are reported as means±SD. Comparison between two groups was determined by parametric, unpaired, two-tailed *t*-test. P<0.05 was considered significant.

## Results

### Establishment of EAN model

Mice presented with apparent EAN symptoms from day 8 to 28 after immunization compared to mice without immunization, and exhibited elevated clinical symptoms scores from day 10 to 28 compared to mice without immunization. Furthermore, after immunization, mice showed increased inflammation and demyelination in sciatic nerves compared to mice without immunization. These results indicated the successful establishment of the EAN model.

### Fasudil decreased clinical symptoms score

**Figure 1:**
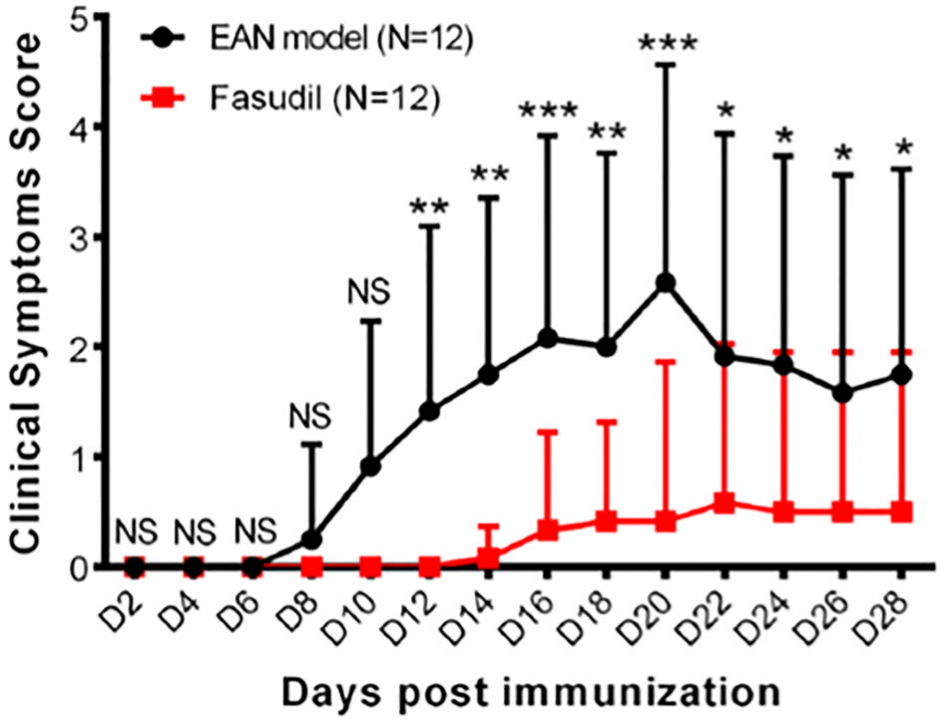
Clinical symptoms score at each assessment after induction of experimental autoimmune neuritis (EAN) in mice treated with fasudil and control (EAN model). Data are reported as mean and SD. *P<0.05, **P<0.01, ***P<0.001 compared to the EAN model (*t*-test). NS, not significant.

Clinical symptoms score was attenuated in the Fasudil group compared to the EAN model group from day 12 to 28 (all P<0.05, Figure 1).

### Fasudil reduced inflammatory cell infiltration and demyelination of sciatic nerves

**Figure 2:**
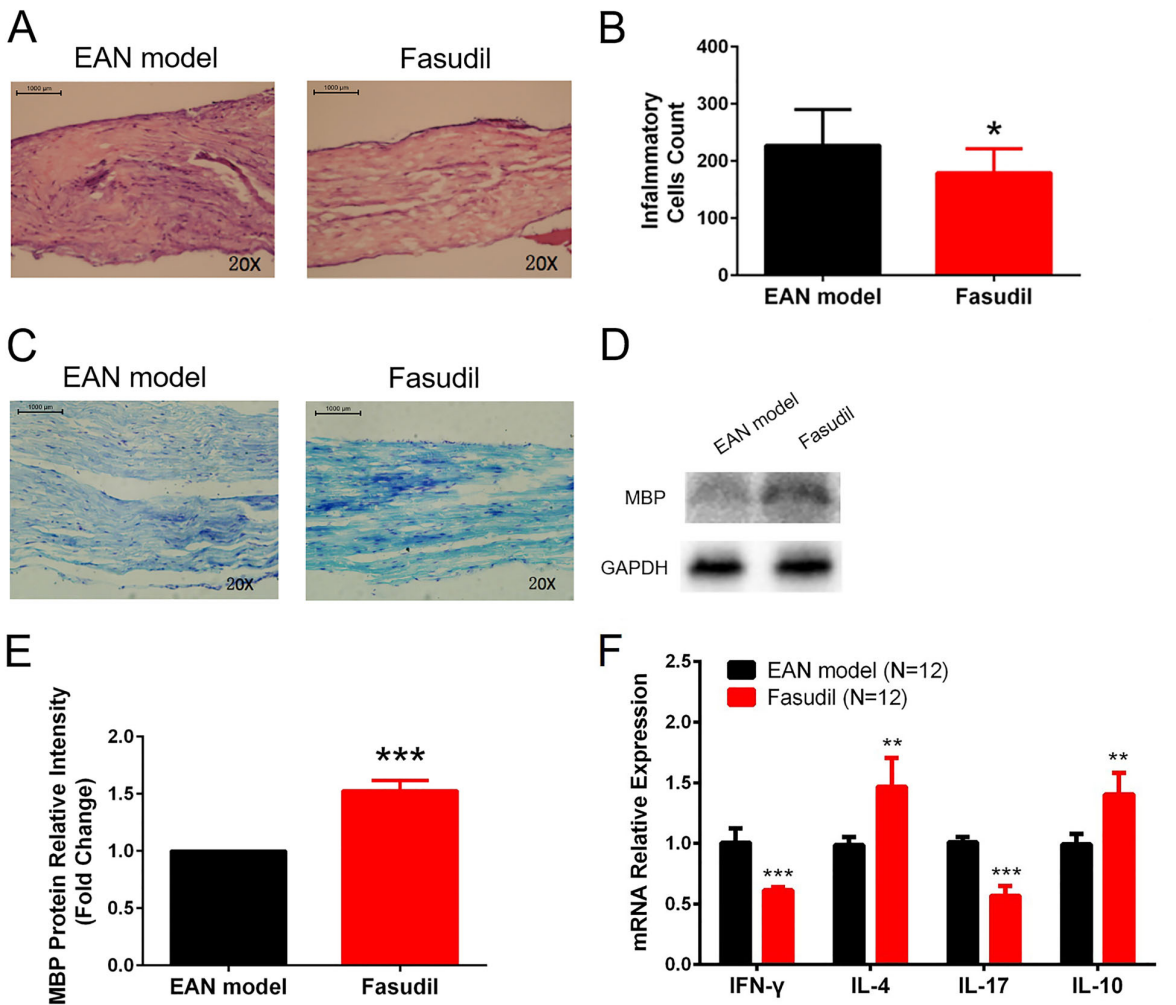
Sciatic nerves inflammation and demyelination in experimental autoimmune neuritis (EAN) mice treated or not with fasudil. Fasudil decreased inflammatory cell infiltration in sciatic nerves (**A** and **B**), and attenuated sciatic nerves demyelination at Day 28 (**C**). Scale bars: 1000 μm. **D** and **E**, Fasudil increased sciatic nerves anti-myelin basic protein (MBP) expression at day 28. **F**, Fasudil decreased interferon (IFN)-γ and interleukin (IL)-17 mRNA expressions and increased IL-4 and IL-10 mRNA expressions at day 28. Data are reported as mean and SD. *P<0.05, **P<0.01, ***P<0.001 compared to the EAN model (*t*-test).

HE staining disclosed that inflammatory cell count in sciatic nerves was decreased in the Fasudil group compared to the EAN model group at day 28 (P<0.05, Figure 2A and B). Luxol fast blue staining revealed that demyelination of sciatic nerves was attenuated in the Fasudil group (Figure 2C), while MBP expression in sciatic nerves was increased (P<0.001) (Figure 2D and E). IFN-γ (P<0.001) and IL-17 (P<0.001) mRNA expressions were decreased, while IL-4 (P<0.01) and IL-10 (P<0.01) mRNA expressions were increased in the Fasudil group (Figure 2F). These results indicated that fasudil reduced inflammation and demyelination of sciatic nerves.

### Fasudil promoted M2 macrophage polarization in sciatic nerves

**Figure 3:**
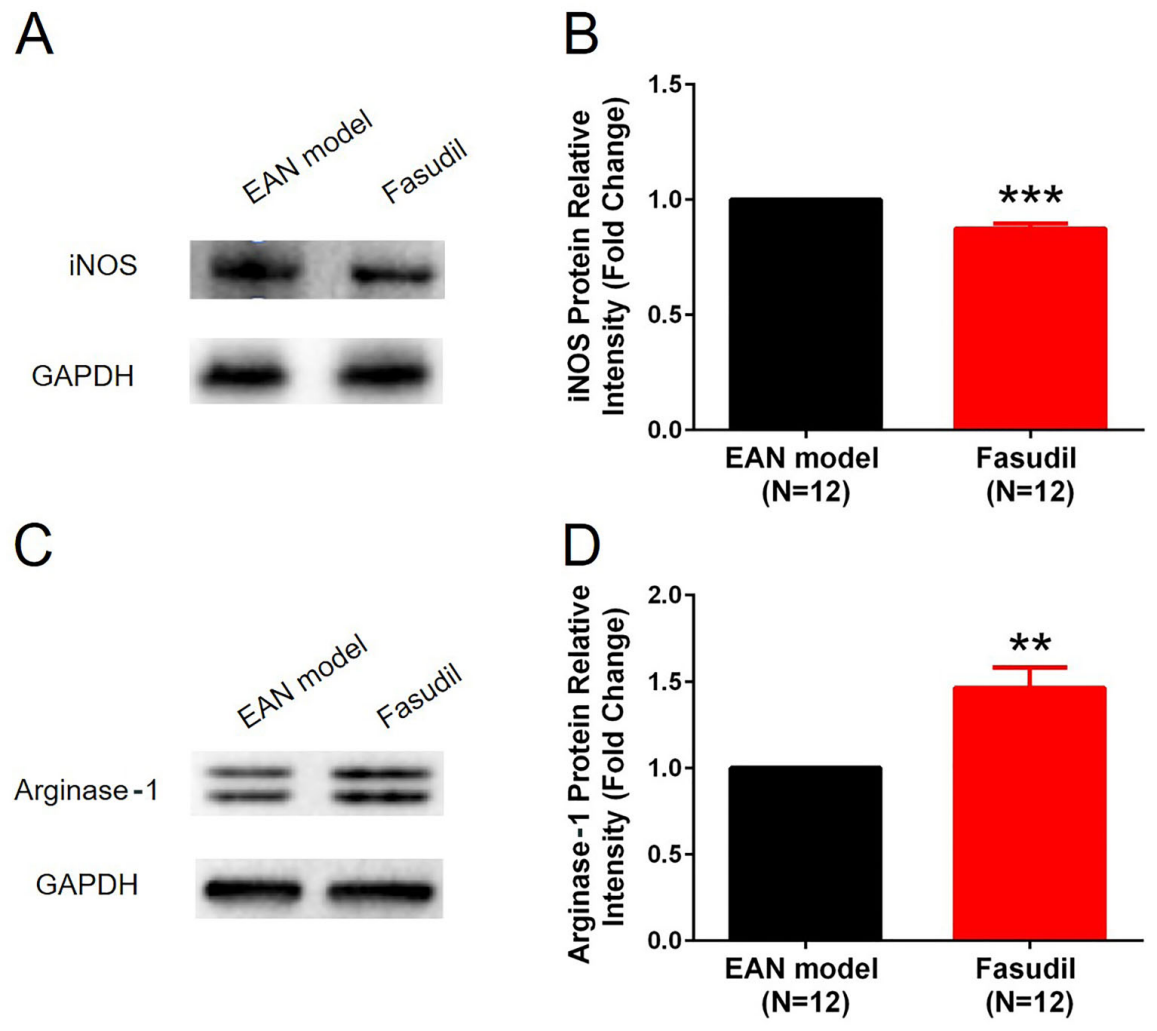
Inducible nitric oxide synthase (iNOS) and arginase-1 protein expressions after induction of experimental autoimmune neuritis (EAN). Fasudil reduced M1 macrophages marker iNOS protein expression (**A** and **B**) and enhanced M2 macrophages marker arginase-1 expression (**C** and **D**) in sciatic nerves at day 28. Data are reported as mean and SD. **P<0.01, ***P<0.001 compared to the EAN model (*t*-test).

In order to investigate the effect of fasudil on regulating macrophages activation, M1 macrophages marker iNOS and M2 macrophages marker arginase-1 were measured in sciatic nerves at Day 28. iNOS protein expression was decreased (P<0.001, Figure 3A and B) while arginase-1 protein expression was increased (P<0.01, Figure 3C and D) in the Fasudil group compared with the EAN model group. These results indicated that fasudil promoted M2 macrophage polarization in sciatic nerves.

### Fasudil decreased serum anti-P0 peptide 180-199 IgG level

**Figure 4:**
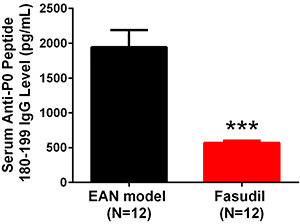
Serum anti-P0 peptide 180–199 immunoglobulin G (IgG) level after induction of experimental autoimmune neuritis (EAN). Fasudil decreased serum anti-P0 peptide 180–199 IgG level at day 28. Data are reported as mean and SD. ***P<0.001 (*t*-test).

To measure antigen specific humoral immune response, serum anti-P0 peptide 180–199 IgG level was determined at day 28, which was greatly decreased in the Fasudil group compared with the EAN model group (P<0.001, Figure 4).

### Fasudil reduced Th1 and Th17 proportions in splenocytes

**Figure 5:**
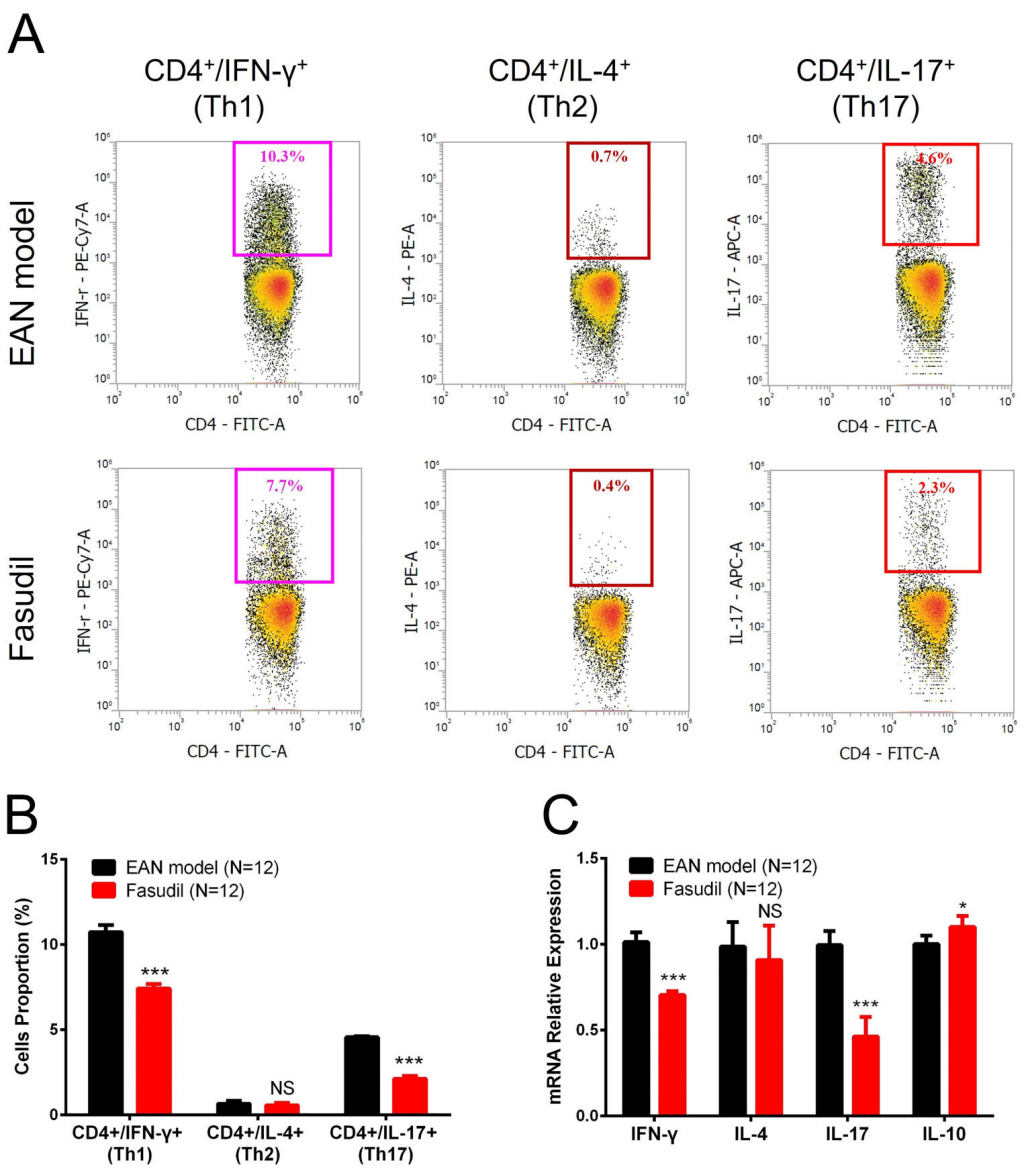
Th1, Th2, and Th17 cell proportion in splenocytes after induction of experimental autoimmune neuritis (EAN). Fasudil decreased CD4^+^IFN-γ^+^ cell proportion and CD4^+^IL-17^+^ cell proportion, but did not affect CD4^+^IL-4^+^ cell proportion in splenocytes at day 28 (**A** and **B**). Fasudil also reduced interferon (IFN)-γ and interleukin (IL)-17 mRNA expression, elevated IL-10 expression, while did not affect IL-4 mRNA expression (**C**). Data are reported as mean and SD. *P<0.05, ***P<0.001 compared to the EAN model (*t*-test).

CD4^+^IFN-γ^+^ cell proportion (P<0.001) and CD4^+^IL-17^+^ cell proportion (P<0.001) in splenocytes were both decreased in the Fasudil group compared to the EAN model group, while CD4^+^IL-4^+^ cell proportion was similar between the two groups (P>0.05) at day 28 (Figure 5A and B). IFN-γ mRNA (P<0.001) and IL-17 mRNA (P<0.001) expressions were decreased, IL-4 mRNA expression was no different (P>0.05), while IL-10 mRNA expression was increased (P<0.05) in the Fasudil group compared to the EAN model group at day 28 (Figure 5C). These results suggested that fasudil reduced Th1 and Th17 proportions in splenocytes.

### Fasudil elevated Treg proportion in splenocytes

**Figure 6:**
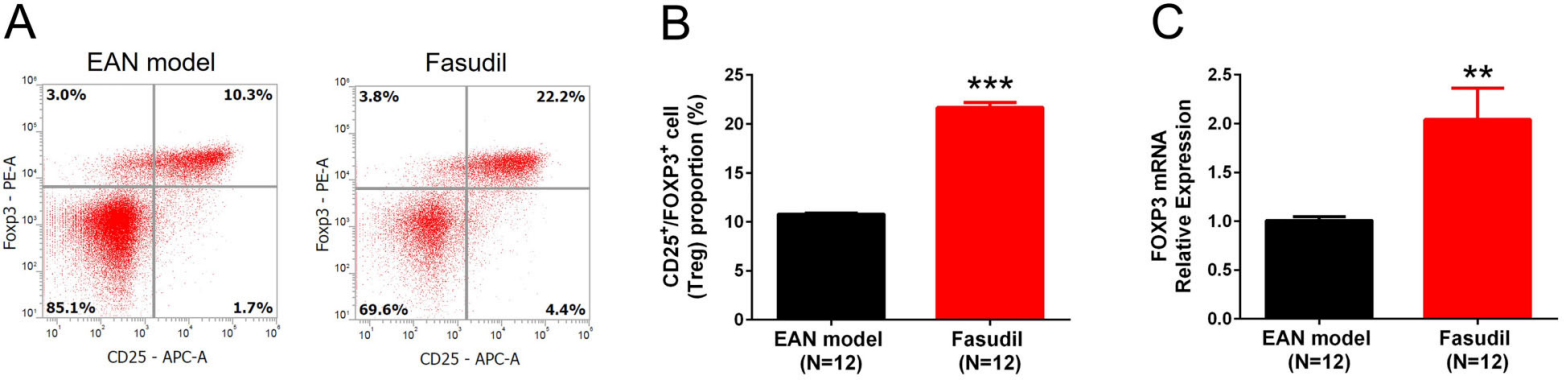
Tregs proportion in splenocytes after induction of experimental autoimmune neuritis (EAN). Fasudil increased CD25^+^FOXP3^+^ cell proportion (**A** and **B**) and FOXP3 mRNA expression (**C**) in splenocytes at day 28. Data are reported as mean and SD. **P<0.01, ***P<0.001 (*t*-test).

CD25^+^FOXP3^+^ cell proportion in splenocytes was increased in the Fasudil group compared to the EAN model group at day 28 (P<0.001, Figure 6A and B). FOXP3 mRNA expression was also higher in the Fasudil group (P<0.01, Figure 6C). These results suggested that fasudil elevated Treg proportion in splenocytes.

## Discussion

In the present study, we observed that in EAN mice: 1) fasudil attenuated clinical symptoms score and serum anti-P0 peptide 180–199 IgG level; 2) fasudil reduced inflammatory cell infiltration as well as demyelination, and promoted M2 macrophage polarization in sciatic nerves; 3) fasudil reduced Th1 and Th17 proportions and elevated Treg proportion in splenocytes.

A study revealed that fasudil induces early functional recovery of spinal cord injury in rats by promoting microglia phagocytosis (15). Another study showed that fasudil promotes normal motor nerve conduction velocity in diabetic rats by assuring the proper localization of adhesion-related molecules in myelinating Schwann cells (16). A recent study reported that fasudil plus bone marrow stromal cells (BMSCs) treatment attenuates Alzheimer disease progression via regulating the peripheral immune system (17). Besides, our previous study found that fasudil attenuated EAE progression via decreasing IL17, IFN-γ/IL-4 ratio, inflammatory cell infiltration, as well as reducing damage of demyelination and axon degeneration (13). Only one recent study has reported that fasudil decreases clinical symptoms and attenuates demyelination as well as axonal degeneration in EAN mice, while the detailed mechanism, especially the regulation of immune cells, is not explored in that study (18).

The possible explanations for our results are as follows: 1) fasudil promoted M2 macrophage polarization in sciatic nerves (decreased M1 macrophage proportion and increased M2 macrophage proportion), leading to decreased inflammation and damage to sciatic nerves, which was verified in our subsequent experiments; 2) fasudil reduced Th1 and Th17 proportions and elevated Treg proportion in splenocytes, thus decreasing systemic accumulating inflammation and related injury, which was exhibited in our subsequent experiments; 3) fasudil acted as a regulator of actin organization, myosin contractility, or other molecule functions, resulting in neuroprotective effect in EAN, however this hypothesis needs further verification.

It is now considered that macrophages can be polarized into M1 type or M2 type. The former exhibits pro-inflammatory effect while the latter presents anti-inflammatory effect, and their dysregulation greatly contributes to the pathogenesis of both GBS and EAN (14,19). It has been reported that M1 macrophages participate in the induction course of EAN as a major factor by promoting cellular cytotoxicity and production of Th1 cytokines, while M2 macrophages increase the content of neurotrophic factors, promoting the repair of myelin sheath and axon regeneration in EAN by inducing the apoptosis of T cells and secreting anti-inflammatory cytokines such as IL-10 and TGF-beta (20,21). Thus, we investigated the effect of fasudil on regulating M1/M2 macrophages in EAN. The results indicated that fasudil might exhibit a therapeutic role in EAN via promoting M2 macrophage polarization.

Th1 and Th17 cells activation are closely involved in the pathology of GBS and its animal model EAN (22,23). As the main marker of Th1 reaction, IFN-γ has a pro-inflammatory role that activates endothelial cells, macrophages, and T cells, and induces other cytokines such as TNF-α, IL-1β, and IL-6 expressions (24,25). In our study, we observed that fasudil decreased the CD4^+^IFN-γ^+^ cell proportion and IFN-γ mRNA expression in splenocytes of EAN mice, which indicated that fasudil might have a protective role in EAN via reducing Th1 cells activation. Furthermore, IL-17 is a key cytokine excreted by Th17 cells, and also serves as a common marker of Th17 cells reaction, which exhibits a pro-inflammatory role that enhances multiple inflammatory signaling pathways such as NF-κB signaling, MAPK signaling, and cytokine-cytokine interaction signaling (26,27). In our study, we observed that fasudil lowered the CD4^+^IL-17^+^ cell proportion and IL-17 mRNA expression in splenocytes of the EAN model, which suggested fasudil might function in EAN via reducing Th17 cells activation.

Dysregulated Th1/Th2 paradigm is also considered a key regulator in GBS and EAN development and progression (28). IL-4, IL-5, IL-10, and IL-13 are the main cytokines excreted by Th2 cells, and neurotrophic factor brain derived neurotrophic factor (BDNF) and NT-3 are also produced by Th2 cells, which play critical roles in GBS and EAN etiology as well (23). Among these factors, IL-4 serves as a key marker of Th2 cell activation, which promotes the differentiation of Th cells to Th2 cells by regulating intrinsic signal factor STAT6, and acts as an anti-inflammatory cytokine as well as a protective factor in GBS and EAN (29–32). In our study, we observed that fasudil did not influence the CD4^+^IL-4^+^ cell proportion or IL-4 mRNA expression in splenocytes of EAN mice, which suggested fasudil had an effect in treating EAN independent of Th2 cells activation.

Tregs are known as regulating T cells, mainly expressing two anti-inflammatory cytokines (IL-10 and TGF-β1), which play important roles in maintaining immune homeostasis (33). Dysregulated Tregs activation is associated with the development and progression of a variety of human autoimmune diseases including GBS (34,35). For instance, CD4^+^CD25^+^Foxp3^+^ Tregs are decreased in the acute phase of GBS (34); Tregs are also reduced in peripheral nerves during the acute phase of EAN, while after treatment, the number of Tregs is increased (35). In our study, fasudil elevated the CD25^+^FOXP3^+^ cell proportion and FOXP3 mRNA expression in splenocytes of EAN mice, which suggested that it might present a protective role in EAN via inducing Tregs activation.

In conclusion, fasudil treatment of EAN mice attenuated clinical symptoms, nerve inflammatory cell infiltration, and demyelination, promoted nerve M2 macrophage polarization, and reduced Th1/Th17 cells while it elevated Tregs among splenocytes. These results provided evidence for the future application of fasudil in treating GBS patients in clinical practice.
